# Identification of Target Gene and Interacting Protein of Two *LaSCL6* Alternative Splicing Variants Provides Novel Insights into Larch Somatic Embryogenesis

**DOI:** 10.3390/plants13213072

**Published:** 2024-10-31

**Authors:** Qiao-Lu Zang, Zha-Long Ye, Li-Wang Qi, Wan-Feng Li

**Affiliations:** 1 State Key Laboratory of Tree Genetics and Breeding, Key Laboratory of Tree Breeding and Cultivation of the National Forestry and Grassland Administration, Research Institute of Forestry, Chinese Academy of Forestry, Beijing 100091, China; zangql@sxau.edu.cn (Q.-L.Z.); kemiye@caf.ac.cn (Z.-L.Y.); lwqi@caf.ac.cn (L.-W.Q.); 2 College of Horticulture, Shanxi Agricultural University, Jinzhong, 030801, China

**Keywords:** Japanese larch, miR171, *LaSCL6*, somatic embryogenesis, regulatory network

## Abstract

Somatic embryogenesis is valuable for clonal propagation and genetic improvement, and it also serves as an ideal system for studying plant development mechanisms. In *Larix kaempferi*, microRNA171 and its target gene *L. kaempferi SCARECROW-LIKE6* (*LaSCL6*), which has two alternative splicing variants, can regulate somatic embryogenesis; however, the underlying molecular mechanism is still unknown. In this study, we overexpressed these two *LaSCL6* variants in *Oryza sativa* and *Arabidopsis thaliana* and then used the RNA-Seq method to screen genes from *O. sativa* and *A. thaliana*, whose expression patterns are related to those of *LaSCL6* variants. The screened genes were then used to search *L. kaempferi* proteins to identify the candidate target genes of *LaSCL6*. After yeast one-hybrid and dual- luciferase transcriptional activity assays, *cytochrome P450*, *family 89*, *subfamily A*, *polypeptide 5* (*CYP89A5*), and *wall-associated receptor kinase-like 20* (*WAKL20*) were confirmed to be the target genes of *LaSCL6-var1*; in addition, *WAKL20* and *UDP-glycosyltransferase 85A3* (*UGT85A3*) were confirmed to be the target genes of LaSCL6-var2. Moreover, APETALA2-like protein 2, a transcription factor from the AP2/ERF family, was shown to interact with LaSCL6-var1 and LaSCL6-var2. Taken together, our results suggest a regulatory network of miR171-*LaSCL6*. The findings presented here not only provide novel insights into the regulation of the miR171-*LaSCL6* module but also explain the mechanism underlying larch somatic embryogenesis and other biological processes.

## 1. Introduction

Somatic embryogenesis is a valuable technique in the clonal propagation and genetic improvement of plants, and it plays a critical role in larch breeding programs [1,2,3,4]. The process of conifer somatic embryogenesis is divided into two main stages: pro-embryogenic masses and somatic embryo [5]. In the pro-embryogenic masses stage, the cell passes from a primitive single-cell aggregate to aggregates of several cells and then to cellular clusters; these pro-embryogenic masses then differentiate to form a somatic embryo, which is a bipolar structure that has no vascular connection with the maternal tissue [6,7]. Somatic embryogenesis is regulated by a number of external and internal factors; these include genotype, explant status, plant growth regulators, and sources of carbon and nitrogen [8,9]. The induction of embryogenic cultures and the maintenance of embryogenic potential are both important for somatic embryogenesis; however, the occurrence of non-embryogenic cultures limits its efficient utilization [5,10,11]. Further studies of the molecular basis of these processes are therefore required to better understand and improve this technique.

MicroRNA affects somatic embryogenesis by regulating its target gene at the post-transcription level [12,13,14,15]. The miR171 family is highly conserved and functions via regulation of its target gene *SCARECROW-LIKE6* (*SCL6*, also known as *HAIRY MERISTEM* or *LOST MERISTEMS*), which is a transcription factor from the GRAS (GAI-RGA-SCR) family [16,17,18]. In citrus, lily, and larch, miR171-*SCL6* takes part in the development of embryogenic cultures and in the maintenance of embryogenic potential [10,19,20,21]. However, the molecular mechanism by which miR171-*LaSCL6* regulates larch somatic embryogenesis has not yet been elucidated.

The molecular mechanisms of *SCL6* have been demonstrated in the regulation of other biological processes. For example, in *Arabidopsis thaliana*, SCL6 interacts with WUSCHEL (WUS) to regulate meristem through the CLAVATA3-WUS pathway [22,23] and interacts with SQUAMOSA promoter-binding-like protein (SPL) to control flowering and trichome initiation [24]. LbrSCL6 also interacts with WUSCHEL-related homeobox4 (LbrWOX4) to regulate maturation in *Lilium* [25]. In addition, SCL6 interacts with the DELLA protein, and their interaction reduces the binding activity of SCL6 to the promoter of *protochlorophyllide oxidoreductase C* (*PORC*), which regulates the biosynthesis of chlorophyll [26]. Moreover, in tea plants, CsSCL6-4 directly promotes the expression of four drought-resistance genes, *peroxiredoxin* (*CsPrx*), *short-chain dehydrogenase/reductase* (*CsSDR*), *omega-3 fatty acid desaturase* (*CsFAD7*), and *eceriferum* (*CsCER1*), by binding motifs in their promoter regions in tea plants [27]. With regard to the existence of these molecular mechanisms in *L. kaempferi*, however, nothing is yet known.

Notably, two *LaSCL6* alternative splicing variants exist in *L. kaempferi* and have different expression patterns during *L. kaempferi* somatic embryogenesis [28], adding more complexity to any study of the functional mechanism of SCL6. In addition, no information is yet available regarding the target gene and interacting protein of LaSCL6. In the present study, we aimed to identify the target genes and interacting proteins of two LaSCL6 alternative splicing variants to provide more information about the functional mechanism of SCL6. The results presented here further enhance our understanding of somatic embryogenesis.

## 2. Results

### 2.1. Thirty Larix Genes Were Screened as the Candidate Target Genes of LaSCL6 after Analyzing the Transcriptomic Responses of O. sativa and A. thaliana to LaSCL6 Overexpression

Given the challenging nature of obtaining transgenic larch plants and the conservative regulation of genes between species, the over-expression vectors of *LaSCL6-var1* and *LaSCL6-var2* were separately constructed and transformed into *O. sativa* and *A. thaliana*. Transcriptome sequencing of wild-type (WT) and transgenic plants was then performed (Parts 1 and 3 in Supplementary Table S1).

**Figure 1:**
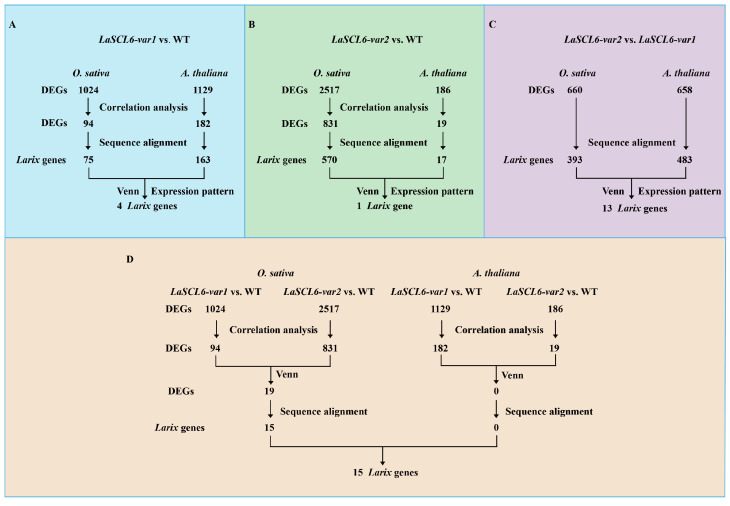
Flow chart of transcriptome analysis. WT, wild-type; DEGs, differentially expressed genes. (**A**) Four *Larix* genes were obtained via expression pattern analysis and sequence alignment of the differentially expressed genes in in the comparison of *LaSCL6-var1* vs. WT in *O. sativa* and *A. thaliana*. (**B**) One *Larix* gene was obtained via expression pattern analysis and sequence alignment of the differentially expressed genes in the comparison of *LaSCL6-var2* vs. WT in *O. sativa* and *A. thaliana*. (**C**) 13 *Larix* genes were obtained via expression pattern analysis and sequence alignment of the differentially expressed genes in the comparison of *LaSCL6-var2* vs. *LaSCL6-var1* in *O. sativa* and *A. thaliana*. (**D**) 15 *Larix* genes were obtained via expression pattern analysis and sequence alignment of the differentially expressed genes in the comparison of *LaSCL6-var1/2* vs. WT in *O. sativa* and *A. thaliana*.

After different comparisons, the differentially expressed genes (DEGs) were obtained (Parts 2 and 4 in Supplementary Table S1). Totals of 1024 and 1129 DEGs were identified in the comparison of *LaSCL6-var1* vs. WT in *O. sativa* and *A. thaliana*, respectively (Figure 1A); totals of 2517 and 186 DEGs were identified in the comparison of *LaSCL6-var2* vs. WT in *O. sativa* and *A. thaliana*, respectively (Figure 1B); and totals of 660 and 658 DEGs were identified in the comparison of *LaSCL6-var2* vs. *LaSCL6-var1* in *O. sativa* and *A. thaliana*, respectively (Figure 1C). In addition, the DEGs in the comparisons of *LaSCL6-var1* vs. WT and *LaSCL6-var2* vs. WT in both *O. sativa* and *A. thaliana* were also identified as potential target genes (Figure 1D).

Values for the Pearson correlation coefficient (PCC) between the expression patterns of the DEGs and *LaSCL6-var1* or *LaSCL6-var2* were calculated; those DEGs with a PCC value of ≥0.9 or ≤−0.9 were considered to have the same or opposite expression pattern as *LaSCL6-var1* or *LaSCL6-var2* and were used for blast analysis.

Totals of 94 and 182 DEGs showed patterns that were the same as or opposite to *LaSCL6-var1* overexpressing *O. sativa* and *A. thaliana*, respectively (Figure 1A); these were then used to blast with *Larix* protein sequences. Ultimately, 75 and 163 *Larix* genes were identified, in which four genes were shared, and the corresponding homologous genes in *O. sativa* and *A. thaliana* had almost the same expression patterns (Figure 1A, Supplemental Table S2).

Totals of 831 and 19 DEGs showed patterns that were the same as or opposite to *LaSCL6-var2* overexpressing *O. sativa* and *A. thaliana*, respectively (Figure 1B); these were then used to blast with *Larix* protein sequences. Ultimately, 570 and 17 *Larix* genes were identified, in which one gene was shared and the corresponding homologous genes in *O. sativa* and *A. thaliana* had almost the same expression patterns (Figure 1B, Supplemental Table S2).

In the comparisons of *LaSCL6-var1* overexpressing *O. sativa* vs. *LaSCL6-var2* overexpressing *O. sativa* and of *LaSCL6-var1* overexpressing *A. thaliana* vs. *LaSCL6-var2* overexpressing *A. thaliana*, 660 and 658 DEGs were obtained, respectively; these were then used to blast with *Larix* protein sequences. Ultimately, 393 and 483 *Larix* genes were identified, in which 13 genes were shared, and the corresponding homologous genes in *O. sativa* and *A. thaliana* had almost the same expression patterns (Figure 1C, Supplemental Table S2).

To identify the potential target genes regulated by both *LaSCL6-var1* and *LaSCL6-var2*, Venn analyses were performed with the DEGs obtained from *O. sativa* and *A. thaliana* after correlation analysis. A total of 19 DEGs were obtained from *O. sativa*, while no DEGs were obtained from *A. thaliana* (Figure 1D). These 19 DEGs were then used to blast with *Larix* protein sequences, and 15 *Larix* genes were ultimately identified (Figure 1D, Supplemental Table S2).

Three genes appear in two comparisons: Larix43329 (identified in the comparison of *LaSCL6-var2* vs. *LaSCL6-var1* and *LaSCL6-var1* vs. WT), Larix12010 (identified in the comparison of *LaSCL6-var1/2* vs. WT and *LaSCL6-var2* vs. *LaSCL6-var1*), and Larix22537 (identified in the comparison of *LaSCL6-var1* vs. WT and *LaSCL6 var1/2* vs. WT). Finally, a total of 30 *Larix* genes (Part 1 in Supplementary Table S3) were obtained and considered as the candidate target genes of *LaSCL6-var1* or *LaSCL6-var2*; these corresponded to 39 *O. sativa* genes and 20 *A. thaliana* genes (Parts 2 and 3 in Supplemental Table S3).

### 2.2. Three Candidate Target Genes Were Confirmed to Be Regulated by LaSCL6

We assumed that if a gene was controlled by *LaSCL6*, the GRAS binding motif would exist in its promoter sequence. We therefore analyzed the numbers of GRAS binding motifs in the promoter sequences of 30 *Larix* genes, 39 *O. sativa* genes, and 20 *A. thaliana* genes (Supplemental Table S3). The results showed that three *Larix* genes and two *O. sativa* genes had no GRAS binding motif in their promoter sequences, while all *A. thaliana* genes had a GRAS binding motif in their promoter sequences. Therefore, 27 *Larix* genes were used for further study. After cloning, two promoter sequences were not obtained. Finally, the promoter sequences of 25 *Larix* genes were obtained (Supplemental Table S4) and used to test their relationships with *LaSCL6-var1* and *LaSCL6-var2*.

**Figure 2:**
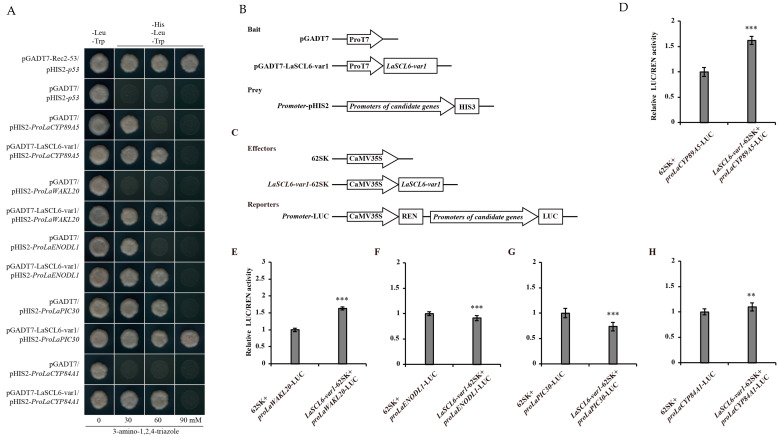
Analysis of interactions between LaSCL6-var1 and its candidate target genes. (**A**) Yeast one-hybrid assays show that LaSCL6-var1 binds to the promoters of *LaCYP89A5*, *LaWAKL20*, *LaENODL1*, *LaPIC30*, and *LaCYP84A1*. (**B**) Schematic diagrams of the bait and prey vectors used in yeast one-hybrid assays. (**C**) Schematic diagrams of the effector and reporter vectors used in dual-LUC assays. (**D**–**H**) Dual-LUC analysis was performed using transient infiltration of *Nicotiana benthamiana* leaves with equal concentrations of *Agrobacterium GV3101* cells transformed with effectors and reporters separately. The values were obtained as a ratio of the activity of firefly luciferase (LUC) and renilla luciferase (REN). Data represent values obtained as mean ± SD of three biological duplications. Error bars represent standard errors. *** *p* ≤ 0.001, ** *p* ≤ 0.01, Student’s *t*-test.

**Figure 3:**
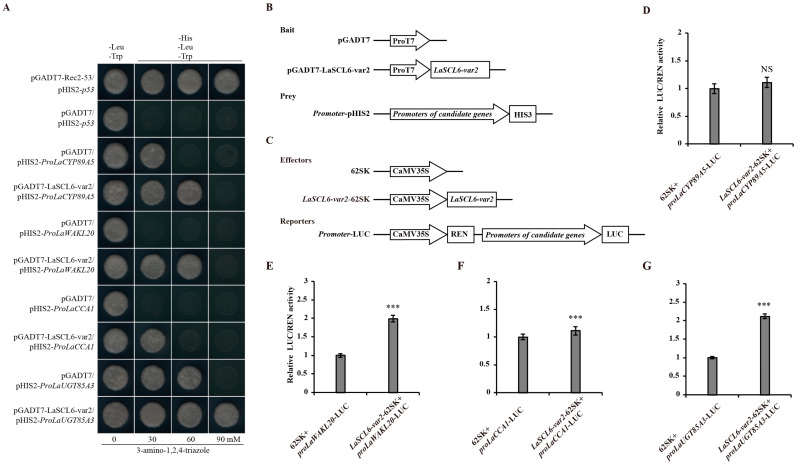
Analysis of interactions between LaSCL6-var2 and its candidate target genes. (**A**) Yeast one-hybrid assays show that LaSCL6-var2 binds to the promoters of *LaCYP89A5*, *LaWAKL20*, *LaCCA1*, and *LaUGT85A3*. (**B**) Schematic diagrams of the bait and prey vectors used in yeast one-hybrid assays. (**C**) Schematic diagrams of the effector and reporter vectors used in dual-LUC assays. (**D**–**G**) Dual-LUC analysis was performed by means of transient infiltration of Nicotiana benthamiana leaves with equal concentrations of *Agrobacterium GV3101* cells transformed with effectors and reporters, respectively. The values were obtained as a ratio of the activity of firefly luciferase (LUC) and renilla luciferase (REN). Data represent values obtained as mean ± SD of three biological duplications. Error bars represent standard errors. *** *p* ≤ 0.001, NS *p* > 0.05, Student’s *t*-test.

A total of 50 yeast one-hybrid (Y1H) assays were performed between LaSCL6-var1 or LaSCL6-var2 and 25 promoters. The relationships between LaSCL6-var1 and *LaCYP89A5*, *LaWAKL20*, *LaENODL1*, *LaPIC30*, and *LaCYP84A1* were confirmed (Figure 2A,B). The relationships between LaSCL6-var2 and *LaCYP89A5*, *LaWAKL20*, *LaCCA1*, and *LaUGT85A3* were also confirmed (Figure 3A,B). Dual-LUC assays further confirmed that LaSCL6-var1 increased the promoter activity of *LaCYP89A5* and *LaWAKL20* (Figure 2C–H) (*p* ≤ 0.05) and LaSCL6-var2 increased the promoter activity of *LaWAKL20* and *LaUGT85A3* (Figure 3C–G) (*p* ≤ 0.05).

### 2.3. LaSCL6-var1 and LaSCL6-var2 Could Interact with LaAP2L2 in the Nucleus

**Figure 4:**
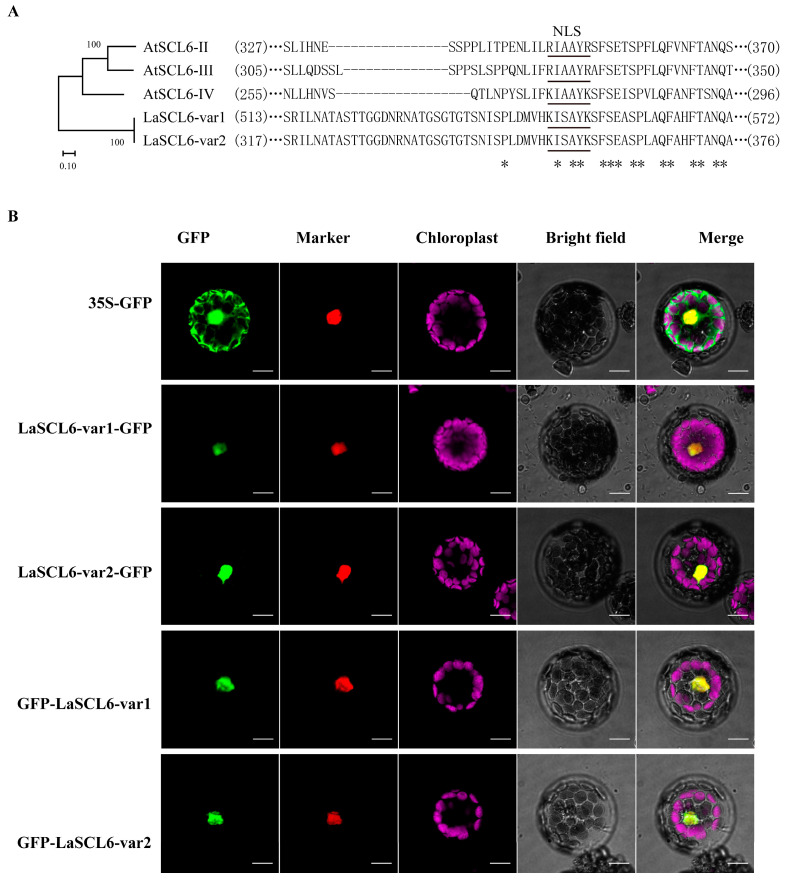
Subcellular localization of LaSCL6-var1 and LaSCL6-var2. (**A**) Alignment and evolutionary analysis of amino acid sequences. The underling amino acid sequences indicate the nuclear localization signal (NLS). * identical sequences. (**B**) Subcellular localization of LaSCL6-var1 and LaSCL6-var2 in *Arabidopsis thaliana* protoplasts. Protoplasts were transiently transformed with 35S:: *LaSCL6-var1*/*LaSCL6-var2*-*GFP* constructs, 35S::*GFP-LaSCL6-var1*/*LaSCL6-var2*, and 35S:: *GFP* vector, respectively. GFP fluorescence was observed with a fluorescence microscope. NLS-mKATE was included for nuclear localization. Images were taken in a dark field for green fluorescence, while the cell outlines were photographed in a bright field. Bars = 10 μm.

LaSCL6-var1 and LaSCL6-var2 both contained the putative nuclear localization signal (NLS) that was also found in AtSCL6 [17] (Figure 4A). Confocal microscopy analysis revealed that LaSCL6-var1-GFP/LaSCL6-var2-GFP and GFP-LaSCL6-var1/GFP-LaSCL6-var2 were localized in the nucleus (Figure 4B).

**Figure 5:**
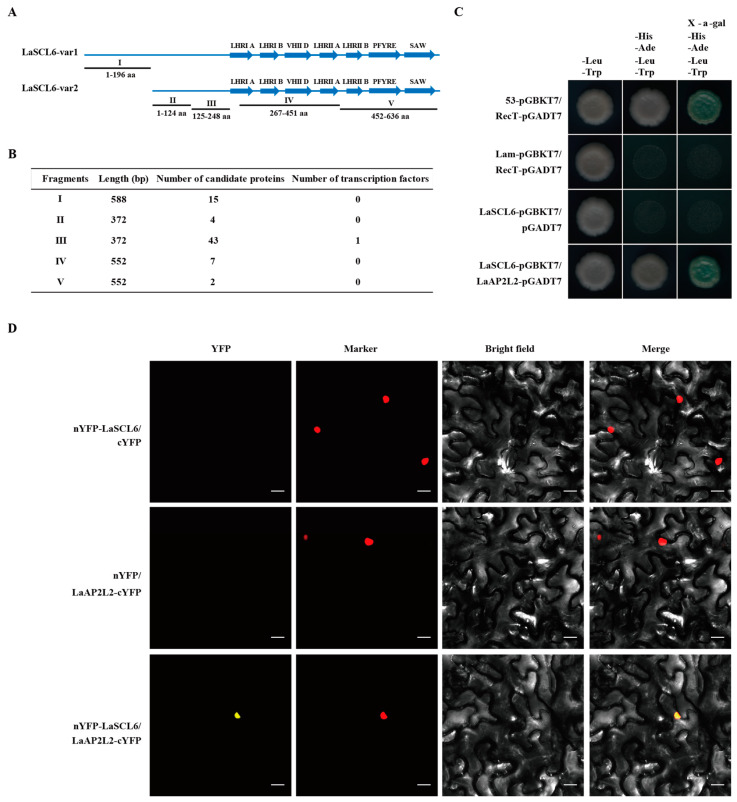
Screening and identification of interaction proteins of LaSCL6-var1 and LaSCL6-var2. (**A**) Schematic of vector construction of yeast two-hybrid screening. (**B**) Statistical results of yeast two-hybrid screening. (**C**) Yeast two-hybrid (Y2H) assays show that LaSCL6 interacts with LaAP2L2. (**D**) Bimolecular fluorescence complementation (BiFC) visualization of the interaction between LaSCL6 and LaAP2L2 in tobacco leaves. YFP fluorescence in nucleus means an interaction. NLS-mKATE was included for nuclear localization. The negative controls were performed using nYFP-LaSCL6 with empty cYFP and LaAP2L2-cYFP with empty nYFP. Bars = 20 μm.

Yeast two-hybrid (Y2H) screen assays in the larch cDNA library were performed using five fragments of LaSCL6 as baits (Figure 5A). The numbers of binding proteins screened by these five fragments were 15, 4, 43, 7, and 2, respectively (Figure 5B). After blast analysis of these 71 sequences, one was annotated as transcription factor encoding an AP2 protein (LaAP2L2, GenBank accession No. KU355275.1) (Figure 5B). Furthermore, this interaction between LaSCL6 and LaAP2L2 was verified via Y2H and BiFC assays (Figure 5C,D).

## 3. Discussion

At present, ChIP-seq is the most effective method for finding the target gene of a transcription factor at the whole-genome level; however, high-quality genomic information and a stable transformation system are essential for the successful application of this method [29]. It is almost impossible to find the target gene of a larch transcription factor using ChIP-seq because the larch genome is large and complex [30,31] and a rapid, efficient and stable larch transformation system is still lacking.

In the present study, a larch transcription factor, *LaSCL6*, was overexpressed in model plants; their transcriptomes were then analyzed to determine the DEGs. Next, following the discovery of homologous genes of these DEGs from larch, and the subsequent determination of their relationships with *LaSCL6*, three target genes (*LaCYP89A5*, *LaUGT85A3*, and *LaWAKL20*) of *LaSCL6* were identified. This method provides a new way to find the target genes of a transcription factor at the whole -genome level in species that are difficult to transform and for which high-quality genomic information is unavailable. In addition, a transcription factor, LaAP2L2, was found to interact with LaSCL6. Taken together, these results help to explain the function and mechanism of *LaSCL6* in larch somatic embryogenesis.

The process of somatic embryogenesis in conifers mainly includes the induction of pro-embryogenic masses, proliferation of embryonic cells, maturation and germination of somatic embryos, and the growth of somatic embryo seedlings [5,11,32]. Consequently, somatic embryogenesis in larch also involves these processes, in which *LaSCL6* and its regulators play important roles.

The induction and proliferation of embryonic cells depend on cell cycle, division, and proliferation. The post-transcriptional regulation of *LaSCL6* by miR171 participates in the regulation of the cell-division mode and the maintenance of embryogenic potential [33]. The heterologous expression of *LaAP2L2* in *Arabidopsis* affects cell proliferation and meristem activity [34]; in the present study, its protein was found to interact with LaSCL6. Likewise, *LaSCL6* target genes might also be involved in the early processes of somatic embryogenesis because their homologous genes have been found to function in the above-mentioned cellular processes. For example, *UGT85U1*, a homologous gene of *LaUGT85A3*, modifies the expression of cell cycle-related genes [35]; in addition, *WAKL* functions in cell wall formation [36], which is a very important part of cell reconstruction. Therefore, if embryogenic cells were induced and proliferated, *LaSCL6* might work in concert with these genes.

The proliferation of embryonic cells and the maturation of somatic embryos are known to occur under conditions of darkness; however, the underlying molecular basis has rarely been studied. In the present study, on the deduction that darkness influences the biosynthesis of chlorophyll, we offered some molecular cues. It is known that *Arabidopsis scl6* mutant plants show increased chlorophyll accumulation [18] and that *SCL6* functions via regulating its target genes, namely *CYP89A9* [37,38], *UGT85A1,* and *UGT85A5* [39,40]. In this study, we also found the involvement of *LaSCL6* regulation of its target genes (*LaCYP89A5* and *LaUGT85A3*) in larch somatic embryogenesis, indicating that *SCL6*-mediated chlorophyll biosynthesis constitutes the molecular basis of darkness treatment of embryonic cultures.

**Figure 6:**
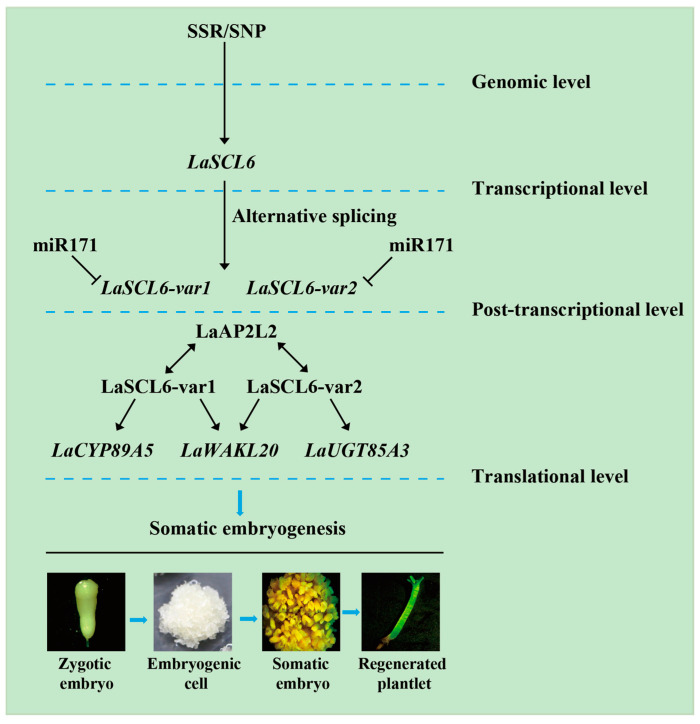
Regulation of *LaSCL6* in larch.

Together, the identified target genes and interacting protein of LaSCL6 help explain the mechanism of the miR171-*LaSCL6* module in larch somatic embryogenesis. In addition, the regulatory network of *LaSCL6* was improved (Figure 6). At the genomic level, simple sequence repeats and single nucleotide polymorphisms influence *LaSCL6* expression [41]. At the post-transcriptional level, *LaSCL6* is regulated by miR171 and the alternative splicing [28,33,42,43]. At the translation level, the interacting protein (LaAP2L2) and target genes (*LaCYP89A5*, *LaWAKL20*, and *LaUGT85A3*) were determined to work together with LaSCL6.

## 4. Materials and Methods

### 4.1. Plant Materials and Growth Conditions

Nipponbare (*O. sativa* L. japonica) seeds, *A. thaliana* ecotype Columbia seeds, *Nicotiana benthamiana* seeds, and immature *L. kaempferi* seeds were used in this study. Nipponbare embryogenic calli were obtained from seeds [44]. *A. thaliana* seeds were sterilized, sown on 1/2 Murashige and Skoog medium, vernalized for 3 d at 4 °C in the dark, and then transferred to a culture room (16 h light/8 h dark, 150 μmol·m^−2^·s^−1^, 22 °C). Seven-day-old uniform *A. thaliana* seedlings were planted in soil, with four plants in each square plastic pot, and transferred to the culture room. *N. benthamiana* seeds were germinated in soil and grown in a growth chamber under controlled conditions (16 h light/8 h dark, 26 °C). Six-week-old *N. benthamiana* plants were used in experiments. Immature *L. kaempferi* seeds were collected from a Dagujia seed orchard (42°22′ N, 124°51′ E) in Liaoning province, in northeast China; from these, the embryonal suspensor mass was generated according to the methods used in a previous study [32].

### 4.2. Vector Construction and Plant Transformation

The full coding sequences of *LaSCL6-var1* (GenBank accession No. MK501379) and *LaSCL6-var2* (GenBank accession No. JX280920) were cloned into the plant expression vector pCAMBIA-1305.1, which contains the cauliflower mosaic virus 35S promoter, using Nco Ⅰ and a Pml Ⅰ restriction sites to generate 35S::*LaSCL6-var1* and 35S::*LaSCL6-var2*, respectively. All constructs were confirmed via sequencing before being introduced into the *Agrobacterium tumefaciens* strains *GV3101* and *EHA105*. All the primers used are listed in Supplemental Table S5.

35S::*LaSCL6-var1* and 35S::*LaSCL6-var2* were separately transformed into Nipponbare by *EHA105* with hygromycin resistance [44]. Transgenic calli and seedlings were screened on a medium containing 50 mg·L^−1^ hygromycin. 35S::*LaSCL6-var1* and 35S::*LaSCL6-var2* were separately transformed into the wild-type *A. thaliana* by *GV3101* using the floral dipping method [45]. Transgenic seeds were screened on 1/2MS plates containing 50 mg·L^−1^ kanamycin. Twenty-one-day-old *A. thaliana* seedlings and thirty-day-old Nipponbare seedlings from four independent lines of the wild type, 35S::*LaSCL6-var1*, and 35S::*LaSCL6-var2* were used for RNA-Seq analysis.

### 4.3. RNA Isolation, RNA-Seq Library Preparation, and Gene Expression Analysis

Total RNA was extracted using an RNAiso Plus reagent kit (TaKaRa, Shiga, Japan). RNA quantity and concentration were measured on a 2100 Bioanalyzer (Agilent, Palo Alto, CA, USA) and NanoDrop ND-1000 (Thermo Scientific, Waltham, MA, USA). Totals of 12 *A. thaliana* and 12 Nipponbare mRNA libraries were prepared according to the Illumina RNA sequencing protocols and sequenced using paired-end sequencing with 150 bp lengths on the NovaSeq 6000 platforms (Illumina, San Diego, CA, USA). The RNA-Seq data have been uploaded to the China National Center for Bioinformation database under the designation PRJCA030648. FastQC (http://www.bioinformatics.babraham.ac.uk/projects/fastqc, accessed on 1 October 2020) and Trimmomatic ver.0.30 [46] were used to perform the initial quality control check of the transcriptome data and produce the clean data, respectively. The clean reads were then separately aligned to the corresponding reference genomes using HISAT2 (v2.1.0) [47]. DEGseq2 was used for differential expression analysis [48]. Genes with the |log_2_ fold change| ≥ 1 and a *p*-value or corrected *p*-value of <0.05 [49] were termed as DEGs. Three pairwise comparisons were performed to identify the DEGs, as follows (1) 35S::*LaSCL6-var1* vs. WT; (2) 35S::*LaSCL6-var2* vs. WT; and (3) 35S::*LaSCL6-var2* vs. 35S::*LaSCL6-var1* (Figure 1).

Common DEGs from the different comparisons were obtained by intersecting the respective gene sets and visualizing using the Venn diagram drawing tool Venny 2.1.0 (https://bioinfogp.cnb.csic.es/tools/venny/index.html, accessed on 29 October 2020). The normalized expression value for each gene was measured in fragments per kilobase of transcript per million mapped reads (FPKM) [50].

### 4.4. Correlation Analysis and Sequence Blast

The FPKM values of *LaSCL6-var1* and *LaSCL6-var2* were calculated for all the sequenced samples. The PCCs between the expression patterns of the DEGs and *LaSCL6-var1* or *LaSCL6-var2* were then analyzed. A PCC value of ≥0.9 or ≤−0.9 was considered to indicate correlation, and the DEG had the same or opposite expression pattern as *LaSCL6-var1* or *LaSCL6-var2*. The best-matching protein sequence for the screened *O. sativa* or *A. thaliana* DEG from the *Larix* genome was identified using BLAST, with an E-value of <10^−5^, and this protein was considered as the potential target gene of LaSCL6. The analysis process is shown in Figure 1.

### 4.5. Promoter Sequence Analysis and Cloning

The promoter sequences of screened genes in *O. sativa* and *A. thaliana* were obtained from EnsemblPlants (http://plants.ensembl.org/index.html, accessed on 29 October 2020). The potential target genes were used to search the *Larix* genome (https://www.ncbi.nlm.nih.gov/nuccore/WOXR00000000.2, accessed on 29 October 2020) [31], and 2000 bp fragments upstream of ATG were regarded as the promoter sequences. Transcription factor binding site prediction was performed using the PlantRegMap server (http://plantregmap.gao-lab.org/binding_site_prediction.php, accessed on 29 October 2020) based on the data of four species, namely, *A. thaliana*, *Populus trichocarpa*, *Solanum lycopersium*, and *Zea mays*, with a *p*-value of <10^−4^ [51]. The GRAS motif numbers in each promoter were then analyzed.

The promoter sequences were amplified from the genomic DNA template and then sequenced. The genomic DNA was isolated with the CTAB plant genome DNA rapid extraction kit (Aidlab Biotech, Beijing China) according to the manufacturer’s protocol. The target fragments were amplified with Platinum^®^ Taq DNA polymerase (Invitrogen, Carlsbad, CA, USA). The PCR products were purified with a gel extraction kit (Tiangen, Beijing China), ligated into the pEASY^®^-T1 simple cloning vector (TransGen, Beijing China), and sequenced. All the primers used are listed in Supplemental Table S5.

### 4.6. Y1H Assay

Y1H assays were performed to verify the prediction results. The open reading frames of *LaSCL6-var1* and *LaSCL6-var2* were amplified by means of PCR and cloned into the pGADT7 vector, resulting in pGADT7-transcription factor plasmids. The sequence fragments of candidate gene promoters were amplified and cloned into the pHIS2 vector, resulting in the pHIS2-promoter plasmids. Next, the bait and prey constructs were co-transformed into the yeast strain Y187 using the lithium acetate method, and yeast cells were plated on SD/-Leu/-Trp media and cultured for 3–5 days. The positive clones were selected and plated separately on SD/-Leu-Trp-His media with 30, 60, or 90 mM 3-amino-1, 2, 4-triazole, and cultured for 3–5 days. Possible interactions between transcription factors and promoters were determined based on the growth status of yeast colonies. All the primers used are listed in Supplemental Table S5.

### 4.7. Dual-LUC Assay

To confirm the Y1H results, Dual-LUC assays were performed. The full-length coding sequences of *LaSCL6-var1* and *LaSCL6-var2* were individually inserted into the pGreenII 0029 62-SK vector to generate the effector constructs. The promoter fragments of candidate genes were individually cloned into pGreenII 0800-LUC vectors to generate the reporter constructs. All recombinant constructs were individually transformed into *A. tumefaciens* strain *GV3101*. *N. benthamiana* leaves were infected with the mixed *Agrobacterium* strain. Detection of fluorescence was performed using the Dual-Luciferase Reporter Assay System (Promega, Madison WI, USA). The LUC activity was normalized to REN activity, and the relative LUC/REN ratios were used to represent the promoter activity. For each combination, LUC/REN ratios from at least three independent transformations were determined. All the primers used are listed in Supplemental Table S5.

### 4.8. Sequence Analysis and Subcellular Localization

Sequences of the GRAS family genes *SCL6-II* (At2G45160), *SCL6-III* (At3G60630), and *SCL6-IV* (At4G00150) were retrieved from the *Arabidopsis* TAIR10 genome release (http://www.arabidopsis.org, accessed on 15 March 2021). The protein sequences of LaSCL6-var1, LaSCL6-var2, and three *A. thaliana* SCL6s were aligned using ClustalX (v2.0). A phylogenetic tree was constructed using a neighbor-joining method with 1000 bootstrap replicates using MEGA (v10.0).

The full-length coding sequences of LaSCL6-var1 and LaSCL6-var2 were separately amplified and inserted at the N- or C-terminal of GFP driven by the CaMV35S promoter. *LaSCL6-var1/LaSCL6-var2-GFP* and *GFP-LaSCL6-var1/LaSCL6-var2* were co-transformed into *A. thaliana* protoplasts using NLS-mKate, which was used as a nuclear marker [52]. Transient expressions of the GFP fusions in *A. thaliana* protoplasts were performed as previously described [53]. Finally, *A. thaliana* protoplasts were observed using confocal microscopy.

### 4.9. Y2H Screening and Identification

The cDNA library for Y2H experiments was constructed by cloning cDNA synthesized from the mRNAs of *L. kaempferi* embryonal-suspensor mass and stems into the prey vector pGADT7 (Takara, Shiga, Japan). The full-length coding sequences of LaSCL6-var1/LaSCL6-var2 and five truncated sequences were amplified via PCR using the indicated primers and inserted into the bait vector pGBKT7 (Takara, Shiga, Japan) using the PEG/LiAc method. These recombinant constructs were introduced into the yeast strain AH109 and tested for autoactivation and toxicity. Because of the strong autoactivation of full-length LaSCL6-var1 and LaSCL6-var2, we used the five truncated fragments for Y2H screening assays, following the user manual of the Matchmaker Gold Y2H system’s user manual (Yeast Protocols Handbook; Takara, Shiga, Japan). The positive clones’ sequencing results were then analyzed via BLAST.

Additional Y2H experiments were performed to test the interactions of the screened transcription factor and the corresponding *LaSCL6* fragment. The coding sequence of the screened transcription factor was cloned into the pGADT7 vector. Next, the constructs were co-transformed into yeast strain AH109, and yeast cells were grown on SD/-Leu-Trp media for 3–5 days. The positive clones were selected and plated onto SD/-Ade/-His/-Leu/-Trp media and cultured for 3–5 days, and positive clones were then transferred onto SD/-Ade/-His/-Leu/-Trp media containing 4 mg·mL^−1^ X-α-Gal to test possible interactions based on the growth status and blue-color development in the yeast colonies. All the primers used are listed in Supplemental Table S5.

### 4.10. BiFC Assay

We used the BiFC assay to directly visualize protein–protein interactions in vivo. The coding sequences of *LaSCL6* and *LaAP2L2* were cloned into the pSM vector to produce the nYFP-LaSCL6 and LaAP2L2-cYFP constructs, respectively. Each construct was individually transformed into *A. tumefaciens* strain *GV3101*. Then, the mixed *Agrobacterium* strain was introduced into *N. benthamiana* leaves via agro-infiltration. After 2 days of incubation, YFP fluorescence was observed in transformed leaf epidermal cells under a laser confocal microscope (Nikon C2-ER, Nikon, Tokyo Japan). All the primers used are listed in Supplemental Table S5.

## 5. Conclusions

In the present study, by identifying the target genes and interacting protein of *LaSCL6* in *L. kaempferi*, we improved the regulatory network of *LaSCL6* (Figure 6), in which *LaSCL6* is regulated at several levels. Analyses of this regulatory network help to increase our understanding of the molecular mechanism of miR171-*LaSCL6* in larch somatic embryogenesis.

## Data Availability

Sequence data and corresponding GenBank accession numbers can be found in Supplemental Table S4. The RNA-Seq data have been uploaded to the China National Center for Bioinformation database under the designation PRJCA030648.

## Supplementary Materials

The following supporting information can be downloaded at: https://www.mdpi.com/article/10.3390/plants13213072/s1, Supplemental Table S1. Summary of transcriptome sequencing of transgenic *Oryza sativa* and *Arabidopsis thaliana*. Supplemental Table S2. Screening and comparison of differentially expressed genes in different comparisons. Supplemental Table S3. GRAS binding sites analysis in predicted gene promoters using information of the four species. Supplemental Table S4. Accession numbers for cloned sequences. Supplemental Table S5. Primes used in the research.

## References

[1] Dobrowolska I., Businge E., Abreu I.N., Moritz T., Egertsdotter U. (2017). Metabolome and transcriptome profiling reveal new insights into somatic embryo germination in Norway spruce (*Picea abies*). Tree Physiol..

[2] Fan Y., Li W., Li Z., Dang S., Han S., Zhang L., Qi L. (2021). Examination of the transcriptional response to *LaMIR166a* overexpression in *Larix kaempferi* (Lamb.) Carr. Biology.

[3] Kang Y., Li W., Zhang L., Qi L. (2021). Over-expression of the cell-cycle gene *LaCDKB1;2* promotes cell proliferation and the formation of normal cotyledonary embryos during *Larix kaempferi* somatic embryogenesis. Genes.

[4] Park M.E., Goryachkina O.V., Tretyakova I.N.M., Muratova E.N. (2023). Cytogenetic characteristics of embryogenic cell lines of different ages obtained by somatic embryogenesis in *Larix sibirica* Ledeb. Contemp. Probl. Ecol..

[5] Klimaszewska K., Hargreaves C., Lelu-Walter M.A., Trontin J.F. (2016). Advances in conifer somatic embryogenesis since year 2000. In Vitro Embryogenesis in Higher Plants Methods.

[6] Siddiqui Z.H., Abbas Z.K., Ansari M.W., Khan M.N. (2019). The role of miRNA in somatic embryogenesis. Genomics.

[7] Wojcik A.M. (2020). Research tools for the functional genomics of plant miRNAs during zygotic and somatic embryogenesis. Int. J. Mol. Sci..

[8] Yang L., Bian L., Shen H.L., Li Y.H. (2013). Somatic embryogenesis and plantlet regeneration from mature zygotic embryos of Manchurian ash (*Fraxinus mandshurica* Rupr.). Plant Cell Tiss. Org..

[9] Sumeera S., Ghori N., Hyat F., Li Y., Chen C. (2023). Use of auxin and cytokinin for somatic embryogenesis in plant: A story from competence towards completion. Plant Growth Regul..

[10] Zhang S.G., Zhou J., Han S.Y., Yang W.H., Li W.F., Wei H.L., Li X.M., Qi L.W. (2010). Four abiotic stress-induced miRNA families differentially regulated in the embryogenic and non-embryogenic callus tissues of *Larix leptolepis*. Biochem. Bioph. Res. Co..

[11] Zhu T., Wang J., Hu J., Ling J. (2022). Mini review: Application of the somatic embryogenesis technique in conifer species. For. Res..

[12] Long J.M., Liu C.Y., Feng M.Q., Liu Y., Wu X.M., Guo W.W. (2018). miR156-SPL modules regulate induction of somatic embryogenesis in citrus callus. J. Exp. Bot..

[13] Li H., Wang J., Yan R., Wang C., Sun H. (2021). Functional characterization of the MiR171a promoter and endogenous target mimics identification in *Lilium pumilum* DC. Fisch. during somatic embryogenesis. Plant Cell Tiss. Org..

[14] Zhang J., Yang Y., Wang Z., Li X., Sun H. (2021). Evidence of the regulatory roles of candidate miRNAs during somatic embryogenesis in Lilium davidiivar.unicolor. J. Plant Growth Regul..

[15] Feng M.Q., Nan J., Wang P.B., Liu Y., Xia Q.M., Jia H.H., Shi Q.F., Long J.M., Xiao G.A., Yin Z.P. (2023). miR171-targeted *SCARECROW-LIKE* genes *CsSCL2* and *CsSCL3* regulate somatic embryogenesis in citrus. Plant Physiol..

[16] Pysh L.D., Wysocka-Diller J.W., Camilleri C., Bouchez D., Benfey P.N. (1999). The GRAS gene family in Arabidopsis: Sequence characterization and basic expression analysis of the *SCARECROW-LIKE* genes. Plant J..

[17] Llave C., Kasschau K.D., Rector M.A., Carrington J.C. (2002). Endogenous and silencing-associated small RNAs in plants. Plant Cell.

[18] Wang L., Mai Y.X., Zhang Y.C., Luo Q., Yang H.Q. (2010). MicroRNA171c-targeted *SCL6-II*, *SCL6-III*, and *SCL6-IV* genes regulate shoot branching in *Arabidopsis*. Mol. Plant.

[19] Wu X.M., Kou S.J., Liu Y.L., Fang Y.N., Xu Q., Guo W.W. (2015). Genomewide analysis of small RNAs in nonembryogenic and embryogenic tissues of citrus: microRNA- and siRNA-mediated transcript cleavage involved in somatic embryogenesis. Plant Biotechnol. J..

[20] Li H., Zhang J., Yang Y., Jia N., Wang C., Sun H. (2017). miR171 and its target gene *SCL6* contribute to embryogenic callus induction and torpedo-shaped embryo formation during somatic embryogenesis in two lily species. Plant Cell Tiss. Org..

[21] Shi Q.F., Long J.M., Yin Z.P., Jiang N., Feng M.Q., Zheng B., Guo W.W., Wu X.M. (2022). miR171 modulates induction of somatic embryogenesis in citrus callus. Plant Cell Rep..

[22] Zhou Y., Yan A., Han H., Li T., Geng Y., Liu X., Meyerowitz E. (2018). HAIRY MERISTEM with WUSCHEL confines CLAVATA3 expression to the outer apical meristem. Science.

[23] Zhou Y., Liu X., Engstrom E.M., Nimchuk Z.L., Pruneda-Paz J.L., Tarr P.T., Yan A., Kay S.A., Meyerowitz E.M. (2014). Control of plant stem cell function by conserved interacting transcriptional regulators. Nature.

[24] Xue X.Y., Zhao B., Chao L.M., Chen D.Y., Cui W.R., Mao Y.B., Wang L.J., Chen X.Y. (2014). Interaction between two timing microRNAs controls trichome distribution in Arabidopsis. PLoS Genet..

[25] Li Q., Song M., Wang Y., Lu P., Ge W., Zhang K. (2024). Unraveling the molecular mechanisms by which the miR171b-*SCL6* module regulates maturation in *Lilium*. Int. J. Mol. Sci..

[26] Ma Z., Hu X., Cai W., Huang W., Zhou X., Luo Q., Yang H., Wang J., Huang J. (2014). *Arabidopsis* miR171-targeted scarecrow-like proteins bind to GT cis-elements and mediate gibberellin-regulated chlorophyll biosynthesis under light conditions. PLoS Genet..

[27] Tian C., Zhou C., Wen S., Yang N., Tan J., Zhang C., Jiang L., Zheng A., Hu X., Lai Z. (2024). csn-miR171b-3p_2 targets CsSCL6-4 to participate in the defense against drought stress in tea plant. Hortic. Plant J..

[28] Zang Q.L., Li W.F., Qi L.W. (2019). Regulation of *LaSCL6* expression by genomic structure, alternative splicing, and microRNA in *Larix kaempferi*. Tree Genet. Genomes.

[29] Jiang Y., Wang M., Zhang R., Xie J., Duan X., Shan H., Xu G., Kong H. (2020). Identification of the target genes of AqAPETALA3-3 (AqAP3-3) in *Aquilegia coerulea* (Ranunculaceae) helps understand the molecular bases of the conserved and nonconserved features of petals. New Phytol..

[30] Sadovsky M., Putintseva Y., Birukov V., Novikova S., Konstantin K. (2016). De Novo Assembly and Cluster Analysis of Siberian Larch Transcriptome and Genome.

[31] Sun C., Xie Y.H., Li Z., Liu Y.J., Sun X.M., Li J.J., Quan W.P., Zeng Q.Y., Van de Peer Y., Zhang S.G. (2022). The Larix kaempferi genome reveals new insights into wood properties. J. Integr. Plant Biol..

[32] Zhang J., Zhang S., Han S., Wu T., Li X., Li W., Qi L. (2012). Genome-wide identification of microRNAs in larch and stage-specific modulation of 11 conserved microRNAs and their targets during somatic embryogenesis. Planta.

[33] Li W.F., Zhang S.G., Han S.Y., Wu T., Zhang J.H., Qi L.W. (2014). The post-transcriptional regulation of *LaSCL6* by miR171 during maintenance of embryogenic potential in *Larix kaempferi* (Lamb.) Carr. Tree Genet. Genomes.

[34] Li A., Yu X., Cao B.B., Peng L.X., Gao Y., Feng T., Li H., Ren Z.Y. (2017). *LkAP2L2*, an AP2/ERF transcription factor gene of *Larix kaempferi*, with pleiotropic roles in plant branch and seed development. Russ. J. Genet..

[35] Ahrazem O., Rubio-Moraga A., Trapero-Mozos A., Climent M.F.L., Gómez-Cadenas A., Gómez-Gómez L. (2015). Ectopic expression of a stress-inducible glycosyltransferase from saffron enhances salt and oxidative stress tolerance in *Arabidopsis* while alters anchor root formation. Plant Sci..

[36] Hou X., Tong H., Selby J., Dewitt J., Peng X., He Z.H. (2005). Involvement of a cell wall-associated kinase, *WAKL4*, in Arabidopsis mineral responses. Plant Physiol..

[37] Christ B., Sussenbacher I., Moser S., Bichsel N., Egert A., Muller T., Krautler B., Hortensteiner S. (2013). Cytochrome P450 CYP89A9 is involved in the formation of major chlorophyll catabolites during leaf senescence in *Arabidopsis*. Plant Cell.

[38] Mach J. (2013). Chlorophyll breakdown branches out: Identification of a major catabolic route involving cytochrome P450 CYP89A9. Plant Cell.

[39] Jin S.H., Ma X.M., Kojima M., Sakakibara H., Wang Y.W., Hou B.K. (2013). Overexpression of glucosyltransferase *UGT85A1* influences trans-zeatin homeostasis and trans-zeatin responses likely through O-glucosylation. Planta.

[40] Sun Y.G., Wang B., Jin S.H., Qu X.X., Li Y.J., Hou B.K. (2013). Ectopic expression of Arabidopsis glycosyltransferase *UGT85A5* enhances salt stress tolerance in tobacco. PLoS ONE.

[41] Zang Q.L., Li X.Y., Qi L.W., Li W.F. (2020). Identification and characterization of *LaSCL6* alleles in *Larix kaempferi* (Lamb.) Carr. based on analysis of simple sequence repeats and allelic expression. Forests.

[42] Zang Q.L., Zhang Y., Han S.Y., Li W.F., Qi L.W. (2021). Transcriptional and post-transcriptional regulation of the miR171-*LaSCL6* module during somatic embryogenesis in *Larix kaempferi*. Trees.

[43] Xing J.X., Zang Q.L., Ye Z.L., Qi L.W., Yang L., Li W.F. (2024). Overexpression of larch *SCL6* inhibits transitions from vegetative meristem to inflorescence and flower meristem in *Arabidopsis thaliana* (L.) Heynh. Plants.

[44] Roy M., Wu R. (2001). Arginine decarboxylase transgene expression and analysis of environmental stress tolerance in transgenic rice. Plant Sci..

[45] Clough S.J., Bent A.F. (1998). Floral dip: A simplified method for *Agrobacterium*-mediated transformation of *Arabidopsis thaliana*. Plant J..

[46] Bolger A.M., Lohse M., Usadel B. (2014). Trimmomatic: A flexible trimmer for Illumina sequence data. Bioinformatics.

[47] Kim D., Paggi J.M., Park C., Bennett C., Salzberg S.L. (2019). Graph-based genome alignment and genotyping with HISAT2 and HISAT-genotype. Nat. Biotechnol..

[48] Love M.I., Huber W., Anders S. (2014). Moderated estimation of fold change and dispersion for RNA-seq data with DESeq2. Genome Biol..

[49] Benjamini Y., Hochberg Y. (1995). Controlling the false discovery rate a practical and powerful approach to multiple testing. J. R. Statist. Soc. B..

[50] Trapnell C., Williams B.A., Pertea G., Mortazavi A., Kwan G., van Baren M.J., Salzberg S.L., Wold B.J., Pachter L. (2010). Transcript assembly and quantification by RNA-Seq reveals unannotated transcripts and isoform switching during cell differentiation. Nat. Biotechnol..

[51] Tian F., Yang D.C., Meng Y.Q., Jin J., Gao G. (2020). PlantRegMap: Charting functional regulatory maps in plants. Nucleic Acids Res..

[52] Shcherbo D., Murphy C.S., Ermakova G.V., Solovieva E.A., Chepurnykh T.V., Shcheglov A.S., Verkhusha V.V., Pletnev V.Z., Hazelwood K.L., Roche P.M. (2009). Far-red fluorescent tags for protein imaging in living tissues. Biochem. J..

[53] Cheong Y.H., Moon B.C., Kim J.K., Kim C.Y., Kim M.C., Kim I.H., Park C.Y., Kim J.C., Park B.O., Koo S.C. (2003). BWMK1, a rice mitogen-activated protein kinase, locates in the nucleus and mediates pathogenesis-related gene expression by activation of a transcription factor. Plant Physiol..

